# The Physician

**Published:** 1879-07

**Authors:** 


### The Bistoury.

ELMIRA, N. Y., JULY, 1879.

The BISTOURY is published Quarterly, upon the 1st of April, July, October and January, at Fifty Cents a year in advance.

### THE PHYSICIAN.

An anonymous writer, in the *Daily Gazette* of this city, of May 20th, attempts a defense of the medical profession against the insinuation of a former contributor to the same journal, [who makes the insulting inquiry—" Do they prefer patients to the public weal ?"] that we cannot permit to pass without our censure.

This defender of the profession evidently, is not himself a practitioner, else would he not measure the aim and desires of the Physician with that of the ordinary trafficker in merchandise.

We protest against the following sentiment and pronounce it a libel upon the cultured and earnest medical man : " They have ( the physicians) families to support, they have sons and daughters to educate, are as emulous of thrift and as greedy of success as we are."

We deny it !—There is not a physican in this city of considerable practice, but that does more charity work, affords more relief from misery and wretchedness, than any two of our wealthiest citizens. He responds to the call of the sick or wounded at any and all hours of the night, never stops to ask whether his fee is secure, but goes upon the bidding, promptly, affording the relief from suffering for which he has been summoned. Indeed, so proverbial has become the physician's reputation for charitable deeds, that the community have learned to expect it of him. Should he refuse to rise from his comfortable bed any stormy night and obey a call to the bedside of some poor sufferer, because of no proffered reward, the good people to whose ears the unheard of intelligence should come, would not be slow to pronounce their condemnation of such an unchristian act. Yet might the same disapproving souls deem it quite right to refuse without sufficient security for future payment, a sack of meal, a ton of coal, or the delay in payment of the last month's rent. The public are too apt to regard the physician's time and prescriptions as less costly than merchandise, when the exact truth is, it is by far the most expensive need that can be furnished to rich or poor. The physician's health and strength are his capital—the "surplus" that not only contributes to the wants of his own family, but also to that of his numerous patrons. Frequent calls upon this capital enfeeble and sometimes exhaust his ability to do, often wrecking his career of usefulness ere it had fairly begun.

When it is remembered that the sum of money required to thoroughly educate a physician and to fit him for his profession would be considered a handsome competency by the average man, his professional advice or visitation cannot be estimated by the cheerfulness with which he *gives* it.

But the next sentence in the communication referred to, is even more offensive : "Do we volunteer advice to our neighbors and friends in order that they may avoid loss? or, do we sometimes, when it will inure to our advantage, let them go on in the way that will be certain to put money in our pockets?"

This question is asked of the layman, yet implies that the physician transacts his professional business much as would a not overscrupulous tradesman. We repel the insinuation and denounce it as an unmanly one. No man of refinement and culture—such as physicians are supposed to be—will harbor a sentiment like that, nor act upon it. No class of men work more earnestly for the public welfare,—seeking to keep the community free from pestilence and disease, than the physicians. To this end they appoint commissions to investigate the causes and means of preventing contagions;—visit remote countries and attend frequent meetings and councils of their professional brethren, that they may obtain the knowledge necessary to protect their respective localities from the visitation of contagious and dangerous diseases. And all this at their own expense! According to the *Gazette* contributor, it were better that he remain at home, permitting the disease to spread, that his professional duties might thereby be multiplied and his income increased.

But what we particularly desire to call the attention of the general public to, is the vast amount of gratuitous and expensive labor that the physician performs. He responds to the wants of the rich and poor alike; at all hours of the day and night, and in all sorts of weather. He sits by the bedside of the sick soothing the pains of the patient and the fears of the family; spends much valuable time in their company that they may be calmed and quieted by his tranquilizing assurances. Rides many weary miles, himself sick from overwork, to prescribe for a poor sufferer, without fee or reward. Dispenses medicines, advice, visitations, upon every hand, lavishly, too often without even a grateful acknowledgment. Somehow, this sort of thing is *expected* of him. You would be shocked to see him do otherwise.

Please remember that something is due your faithful family physician besides the prompt payment of his bill. Thank him too; heartily, sincerely, for the sleepless nights you have caused him while reflecting upon your case, striving to bring it to a successful termination. Thank him for his unselfish interest in you and for the kind words spoken by your bedside. You owe him more than money. Cheer his heart with your gratitude expressed in words.

